# Adaptive evolution of traits for parasitism and pathogen transmission potential in bat flies

**DOI:** 10.1093/nsr/nwae245

**Published:** 2024-07-16

**Authors:** Guangping Huang, Xing Liu, Xin Huang, Chuang Gao, Zhilin Wang, Junxia Li, Xiaocui Wei, Wen-Hua Yu, Yi Wu, Ying Liu, Jiang Feng, Yang Li, Fuwen Wei

**Affiliations:** CAS Key Laboratory of Animal Ecology and Conservation Biology, Institute of Zoology, Chinese Academy of Sciences, Beijing 100101, China; Jiangxi Provincial Key Laboratory of Conservation Biology, College of Forestry, Jiangxi Agricultural University, Nanchang 330045, China; CAS Key Laboratory of Animal Ecology and Conservation Biology, Institute of Zoology, Chinese Academy of Sciences, Beijing 100101, China; University of Chinese Academy of Sciences, Beijing 100049, China; CAS Key Laboratory of Animal Ecology and Conservation Biology, Institute of Zoology, Chinese Academy of Sciences, Beijing 100101, China; University of Chinese Academy of Sciences, Beijing 100049, China; CAS Key Laboratory of Animal Ecology and Conservation Biology, Institute of Zoology, Chinese Academy of Sciences, Beijing 100101, China; University of Chinese Academy of Sciences, Beijing 100049, China; CAS Key Laboratory of Animal Ecology and Conservation Biology, Institute of Zoology, Chinese Academy of Sciences, Beijing 100101, China; University of Chinese Academy of Sciences, Beijing 100049, China; CAS Key Laboratory of Animal Ecology and Conservation Biology, Institute of Zoology, Chinese Academy of Sciences, Beijing 100101, China; University of Chinese Academy of Sciences, Beijing 100049, China; CAS Key Laboratory of Animal Ecology and Conservation Biology, Institute of Zoology, Chinese Academy of Sciences, Beijing 100101, China; University of Chinese Academy of Sciences, Beijing 100049, China; Key Laboratory of Conservation and Application in Biodiversity of South China, School of Life Sciences, Guangzhou University, Guangzhou 510006, China; Key Laboratory of Conservation and Application in Biodiversity of South China, School of Life Sciences, Guangzhou University, Guangzhou 510006, China; Jilin Provincial Key Laboratory of Animal Resource Conservation and Utilization, Northeast Normal University, Changchun 130024, China; Jilin Provincial Key Laboratory of Animal Resource Conservation and Utilization, Northeast Normal University, Changchun 130024, China; CAS Key Laboratory of Animal Ecology and Conservation Biology, Institute of Zoology, Chinese Academy of Sciences, Beijing 100101, China; University of Chinese Academy of Sciences, Beijing 100049, China; CAS Key Laboratory of Animal Ecology and Conservation Biology, Institute of Zoology, Chinese Academy of Sciences, Beijing 100101, China; Jiangxi Provincial Key Laboratory of Conservation Biology, College of Forestry, Jiangxi Agricultural University, Nanchang 330045, China; University of Chinese Academy of Sciences, Beijing 100049, China

**Keywords:** bat fly, bat, host–pathogen interactions, ectoparasites, adaptive evolution, virus–host dynamics, antiviral RNA interference

## Abstract

Deciphering the mechanisms underlying the transmission and spillover of zoonoses from reservoir hosts is essential in preventing future global pandemics. Bat flies—obligate blood-feeding ectoparasites of bats—are known carriers of diverse viruses. Here, we conducted a *de novo* assembly of a chromosome-level genome for the bat fly species *Phthiridium* sp. Comparative genomic analysis unveiled genes associated with specialized traits, such as the loss of eyes and wings, as well as elongated legs, which have adapted to parasitism on the dense fur of bats. Utilizing small RNA sequencing, we identified a spectrum of known and previously unclassified viruses in bat flies. Notably, experimental evidence indicated that bat flies can also feed on mammalian hosts other than bats, suggesting the potential for the spillover of bat-borne viruses. Furthermore, we demonstrated the role of the bat fly's RNA interference pathway in influencing the diversity and evolution of viruses. In summary, this study not only presents a new genome catalog to unveil the evolutionary mechanisms underpinning bat fly parasitism, but also provides a novel research system that can be used to investigate the mechanisms of cross-species transmission of bat-borne viruses and the co-evolution of bats and viruses.

## INTRODUCTION

Bats are recognized as reservoirs for a diverse range of pathogens. Among these, several are zoonotic, indicating their ability to transmit from bats to humans and other species, directly or through intermediate hosts. Notable zoonotic pathogens associated with bats include the rabies virus, coronaviruses, Nipah virus, Hendra virus and filoviruses [1–3]. Gaining a comprehensive understanding of how bats coexist with these pathogens offers valuable insights into strategies for the prevention and management of zoonotic diseases. Blood-feeding arthropods are known to transmit a wide array of pathogens affecting humans and animals, including viruses, bacteria and protozoa, through either biological or mechanical means [4]. Bats host various ectoparasites, such as bat flies, ticks, mites and fleas [5].

Bat flies (Diptera: Hippoboscidae) are specialized parasitic insects, predominantly feeding on bat blood. They are classified into two families: Streblidae and Nycteribiidae. Currently, ∼230 species of Streblidae across 33 genera and 280 species of Nycteribiidae across 11 genera have been identified [6,7]. Nycteribiidae are wingless and spider-like, whereas most Streblidae possess wings, though not always fully developed. All species of Nycteribiidae feature a small head and prolonged legs, with a somewhat dorsoventrally flattened body. The legs protrude from the upper surface of the thorax, and the small and sharp head can be folded back against the thorax while at rest. Bat flies engage in viviparous puparity—a reproductive process akin to that of tsetse flies (Glossinidae)—resulting in a reduced reproductive rate compared with other dipterans that utilize oviposition [6]. The host specificity of bat flies has been a subject of prolonged debate. Some studies propose a high level of host specificity of bat flies, while earlier research argues otherwise. This discrepancy may be attributed to the co-roosting behavior of various bat species and the consequent sharing of parasites [8,9]. Despite the identification of >500 bat fly species, their genomic data remain undocumented, and the phylogenetic relationships among Nycteribiidae, Streblidae and other members of the Hippoboscidae family are still unclear. Bat flies have a short survival time after being removed from bats, which indicates their physiological dependence on their hosts. This dependency, coupled with the absence of genomic information and fundamental research tools, has significantly impeded research progress in this field [10]. Notably, a variety of viruses were found in bat flies, such as dengue virus (Flaviviridae), Lloviu virus (Filoviridae), Kaeng Khoi virus (Perbunyaviridae) and Mahlapitsi virus (Reoviridae) [5,11–14]

Host cells, in turn, have evolved a multitude of defense systems designed to detect and eliminate viral intruders. These defenses include innate and adaptive immunity, apoptosis and autophagy [15]. For instance, in their defense against viral infections, dipterans such as fruit flies and mosquitoes employ RNA interference (RNAi)—a conserved mechanism of gene silencing facilitated by small RNAs [16]. RNAi can be initiated by long double-stranded RNA (dsRNA) originating from viral genomes or replicative intermediates. These dsRNAs are processed by the enzyme Dicer into small interfering RNAs (siRNAs). Subsequently, these siRNAs become integrated into the RNA-induced silencing complex, in which they guide the recognition and cleavage of complementary viral RNAs [17]. Numerous studies have substantiated that the artificial induction of the RNAi pathway using viral-specific siRNAs can influence viral evolution [18,19]. However, it remains unclear whether bat flies possess a functional RNAi pathway that exerts an impact on viral diversity.

While the bat fly serves as a significant ectoparasitic organism, many of its characteristics remain enigmatic. This study focuses the following issues: (i) the genetic mechanisms of specific adaptive traits of bat flies; (ii) whether bat flies specifically bite bats; (iii) the diversity of viruses harbored by bat flies; and (iv) whether bat flies possess a functional RNAi pathway that can serve an antiviral function. This study will provide novel insights into the adaptive evolution of bat flies and the prevention of bat-borne zoonosis.

## RESULTS

### Genomic features of a bat fly, an ectoparasite of *Rhinolophus* bats

Bat ectoparasites were collected from the Zhangjiajie region in Hunan Province. The ectoparasites found on local bats (*Rhinolophus sinicus* and *R. pearsoni*) included Nycteribiidae, Streblidae, ticks and mites (Fig. S1a). Nycteribiidae were the predominant species, consistently with previous findings that Nycteribiidae species are more prevalent in the Eastern Hemisphere [6]. Given their dominance and ecological significance, Nycteribiidae specimens were selected for genome sequencing in this study.

**Figure 1.**
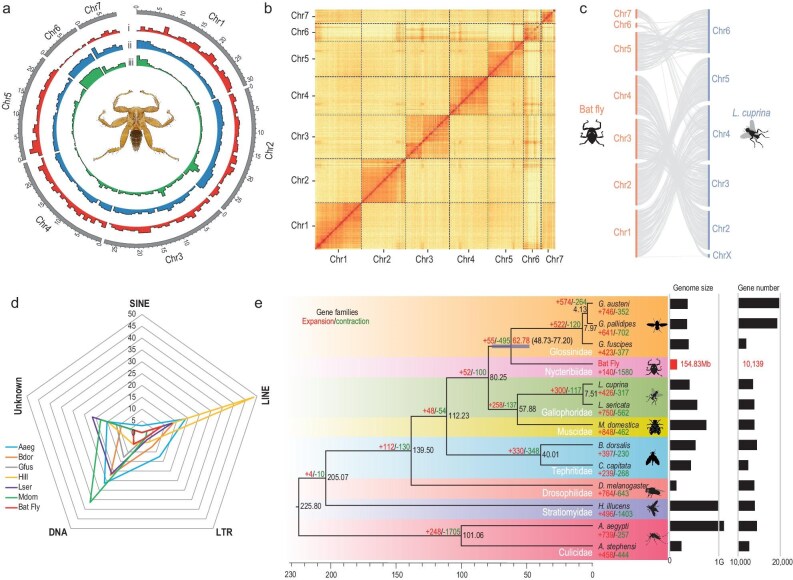
Genomic characterization, phylogenetics and gene family evolution in the bat fly. (a) Genome architecture of the bat fly. A circos plot delineates the genomic features of the bat fly within 1-Mb non-overlapping sliding windows, illustrating (i) the density of the protein-coding genes, (ii) GC content density and (iii) repeat content density. (b) Chromatin interaction profile. Hi–C interaction heat map of bat fly genome. (c) Illustration of the collinear relationship between the protein-coding sequences of the bat fly and *L. cuprina*. (d) Repetitive sequence landscape. Comparative analysis of the composition of repetitive sequences between the bat fly and representative species from the Diptera order. The data are presented as the percentage of each repetitive sequence category relative to the total genome size. Key species abbreviations: Aaeg, *A. aegypti*; Bdor, *B. dorsalis*; Gfus, *G. fuscipes*; Hill, *H. illucens*; Lser, *L. sericata*; Mdom, *M. domestica*. (e) Phylogenetic context and gene family dynamics. Phylogenetic tree encompassing the bat fly and 12 other Diptera species, featuring estimated divergence times and numbers of gene families undergoing expansion and contraction. The 95% confidence intervals for divergence times between the bat fly and Glossinidae species are depicted in brackets and bars. Accompanying histograms display genome sizes and protein-coding gene counts for the respective species.

A chromosome-level reference genome for *Phthiridium* sp. (Diptera: Nycteribiidae) was generated, with a size of 154.83 Mb and contig N50 of 3.92 Mb, which is close to the estimated size of 157.66 Mb of the genome survey that utilized the 17 k-mer distribution (Fig. S1b and Tables S1A–S1C). Ninety-six percent of the assembled sequences were anchored into seven pseudo-chromosomes with a scaffold N50 of 27.70 Mb (Fig. 1a and b, and Table S1A). The 98.34% mapping rate and ×145.18 coverage was obtained by aligning all Illumina clean reads to the assembled genome, respectively, and there was a 94.47% mapping rate and ×327.90 coverage for Nanopore subreaads, respectively (Table S1D). Besides, we identified 966 199 high-quality heterozygous single nucleotide polymorphisms in the sequenced bat fly genome, resulting in a frequency of heterozygous sites of 6.24 × 10^−3^.

The single base accuracy for the assembled genome was estimated to exceed 99.99% (Table S1E). BUSCO assessment showed that 98.83% of conserved insect genes could be found entirely in the assembled genome (Table S1F). In addition, CEGMA evaluation found that 97.98% of core eukaryotic genes were complete (Table S1F). The series of evaluations indicated that the assembly was of high quality. The collinear analysis showed that the pseudo-chromosome 4 of *Phthiridium* sp. displayed a better colinearity with the sex chromosome X of *Lucilia cuprina* (Fig. 1c). Besides, the pseudo-chromosomes 6 and 7 of the bat fly may be the fission of chromosome 4 of the *L. cuprina* (Fig. 1c).

Repeat annotation showed that the repetitive elements covered 28.87% of the genome sequence (Fig. 1d and Table S1G). The repeats proportion of the bat fly, including LINE (long interspersed nuclear elements), SINE (short interspersed nuclear elements), LTR (long terminal repeat) and DNA transposons, is lower than those of other Diptera species, which might result in its extremely smaller genome size (Fig. 1d). A total of 10 139 protein-coding genes were predicted based on combined *de novo* gene prediction, homology-based prediction and transcripts-based prediction (Table S1H), of which 9481 genes were functionally annotated by various databases (Fig. S1c and Table S1I). Statistically, the basic metrics for the annotated genes of the bat fly are consistent with those of related species (Fig. S1d and Tables S1J and S1K). The BUSCO assessment shows that 97.29% of the conserved insect genes were annotated to be complete (Table S1L).

### Comparative genomics of the bat fly, *Phthiridium* sp.

A total of 2589 one-to-one orthologous genes were identified by using OrthoFinder within the bat fly and 12 other Diptera species (Table S1K). The topology of the reconstructed tree was consistent with the previous study (Fig. 1e) [20], with the bootstrap value of all nodes at 100%. The bat fly was classified within the order Nycteribiidae, exhibiting close phylogenetic relationship with species in the order Glossinidae. The divergence time between the bat fly and the species of the genus *Glossina* is estimated to be ∼62.78 Mya [95% highest posterior density (HPD) 48.73–77.20 Mya] (Fig. 1e, Fig. S1e and Table S1M).

**Figure 2.**
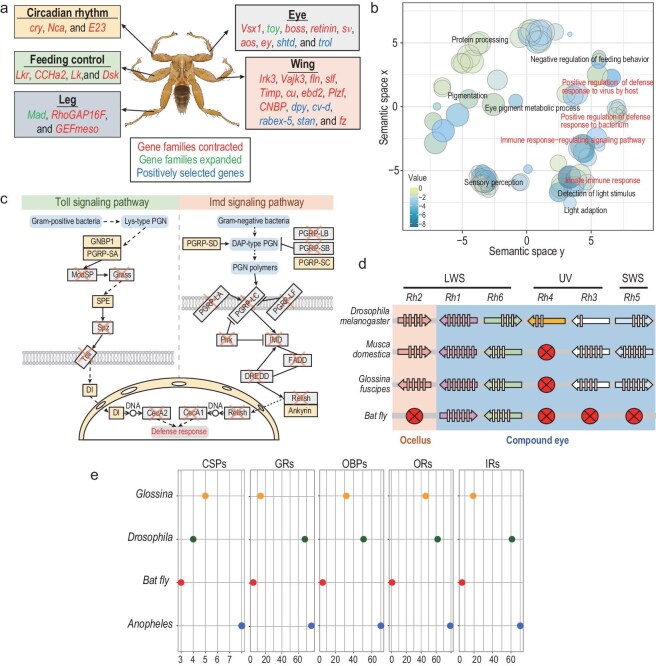
Deciphering genetic mechanisms underlying specialized adaptive traits in the bat fly. (a) Genes with unique evolutionary signals in the bat fly and their putative adaptive function, including circadian rhythm, feeding control and the development of the eye, wing and leg. (b) Results from REVIGO illustrating the contracted gene families within the bat fly genome are presented. ‘Immune’ and ‘non-immune’ related gene ontology (GO) clusters are distinguished by different colors. (c) A schematic representation of the Toll and Imd signaling pathways, focusing on the absence of critical pathway components in the bat fly. Missing genes are annotated with an ‘X’, underlining their absence in the bat fly's genetic make-up. (d) Comparative rhodopsin gene analysis. The gene structures are indicated with rectangles representing exons and the loss of specific genes in a species is marked by a circle. The types of rhodopsins covered include LWS (Long Wavelength Sensitive opsins), UV (UV-sensitive opsins) and SWS (Short Wavelength Sensitive opsins). (e) Gene counts of chemoreceptor genes in genus *Glossina*, the bat fly, *D. melanogaster* and *A. gambiae*. CSPs, chemosensory proteins; GRs, gustatory receptors; OBPs, odorant-binding proteins; ORs, odorant receptors; IRs, ionotropic receptors.

The bat fly genome has 140 significantly expanded and 1580 significantly contracted gene families compared with 12 other species (Fig. 1e). This genome had the largest number of contracted gene families in our selected data set, which was consistent with its dramatic reduction in the number of protein-coding genes. Functional enrichment analyses revealed that the significantly expanded gene families were mainly involved in cell fate specification, the metabolic process, cell differentiation and cell division (Table S2A). For significantly contracted gene families, biological functions including sensory perception, development, immune response, hemostasis and coagulation were enriched (Table S2B). The biological functions of these gene families were manually identified by searching the literature and multiple databases, and it was found that they were associated with eye, wing and leg development, circadian rhythms, feeding behavior and immunity (Fig. 2a and Table S2C).

One hundred and sixty-eight positive selected genes (PSGs) were identified in the bat fly (Table S2D). These genes were overrepresented significantly in Gene Ontology (GO) terms related to the establishment of organelle localization (*P* < 0.001), the pattern specification process (*P* < 0.001) and tube development (*P* < 0.001) (Fig. S2a). In addition, 40 of 168 PSGs remained after conservatively correcting for multiple testing, including genes involved in eye development and vision (*shtd* and *trol*), wing development (*cv-d, dpy, rabex-5* and *stan*) and defense against viruses or bacteria (*hlc* and *tollo*).

### Genes related to immune response

The GO enrichment results showed that these contracted gene families in the bat fly were mainly significantly enriched in immune response-related clusters or pathways (Fig. 2b), such as the innate immune response and positive regulation of the defense response to the virus by the host. In addition, the KEGG enrichment analysis suggests that these contracted immune-related genes were significantly enriched in the Toll/Imd signaling pathway (Fig. 2c). The key regulatory genes in these pathways were lost in the bat fly, including *Grass, Spz, Toll, Pirk, IMD, FADD* and *DREDD*. This pattern has been observed in another obligate blood-feeding hemipteran *Rhodnius prolixus*, which also lost the key genes *Pirk, IMD, FADD* and *DREDD* in the IMD signaling pathway [21].

### Genes related to vision degradation

Arthropods inhabiting dim-light or no-light environments have eyes that have evolved macro- and micro-morphological characteristics following a similar trend to troglobiont species, selectively leading to a reduction in or complete loss of visual systems [22]. Within the suborder Brachycera, *Musca domestica* have neural superposition-type eyes composed of ∼3000 facets, while species in the superfamily Hippoboscidae, which are more closely related to bat flies, only contain hundreds of facets [23]. In particular, cross-species studies of macroscopic morphology showed that the eyes of bat flies contained ≤57 facets [24]. In contrast to the species in the order of Diptera, *Phthiridium* sp. displayed eye degeneration. Genes related to eye development or vision were lost or positively selected in the bat fly (Fig. 2a and Table S2D), such as the loss of the gene *ey* (eyeless) that encodes a transcription factor involving in retinal determination. Mutation in the *ey* results in eye loss in the fruit fly, suggesting that the loss of the gene *ey* in the bat fly may be the main determinant for their eye degeneration [25].

Opsin proteins form complexes with small molecular chromophores and these complexes exhibit sensitivity to specific light wavelengths: Long Wavelength Sensitive (LWS, *Rh1, Rh2* and *Rh6*), Short Wavelength Sensitive (SWS, *Rh5*) and UV (*Rh3* and *Rh4*) [26]. Genomic analysis revealed varying degrees of loss of visual pigment genes. The genome of *Drosophila* species contained six opsin genes, whereas *Glossina*, a genus that is closely related to *Phthiridium*, contained a lineage-specific loss of the dipteran *Rh4* opsins. In this study, we identified two opsin genes (*Rh1* and *Rh6*) in the bat fly (Fig. 2d). Based on the BLAST analysis, the *Rh1* gene is relatively conserved among insects, such as in *D. melanogaster* (79.8% amino acid sequence identity) and in *G. palpalis* (80.0% amino acid sequence identity). Transmembrane domain analysis of the *Rh1* gene predicts that the protein contains seven transmembrane domains, which is a conserved feature of rhodopsin (Fig. S2b). Furthermore, studies have shown that bat fly eyes are functional, as shown by the expression of a *Rh*1 opsin forming a visual pigment [27]. However, *Rh6* has low homology with the genes of other species, with the highest similarity to sequences from *G. palpalis* (40.7% amino acid sequence identity), whose function in the bat fly remains to be determined in future. The bat fly has no ocelli that correspond to the loss of *Rh2* [28]. The loss of the UV and SWS clade rhodopsins (*Rh3*–*Rh5*) in bat flies is consistent with the cave-dwelling environment of their host.

### Genes related to wingless

Among the lost genes, some were associated with wing development (Fig. 2a and Table S2C), such as *Irk3, Vajk3, fln, slf, Timp, cu, ebd2, Plzf, CNBP* and fz, which may have caused wing loss in the bat fly. For example, the gene *Irk3* encodes a protein involved in wing disc development. The gene *slf* (schlaff) encodes a chitin binding protein involved in wing formation and necrotic wing hinges were observed in flies that had downregulated *slf* activity by RNAi [29]. In addition, we identified that the gene *dpy* (dumpy) exhibited positive selection in the bat fly. The *dpy* encodes an extracellular protein that is involved in the aposition of wing surfaces and altering *dpy* expression in developing wings results in predictable changes in wing shapes [30]. Therefore, variations in the above genes might have contributed to the wing loss of the bat fly.

### Genes related to circadian rhythms and metabolic adaptation

Genes associated with circadian rhythms (*cry, Nca* and *E23*) and feeding control (*Lkr, Lk, CCHa2* and *Dsk*) were also detected to be lost in the bat fly (Fig. 2a and Table S2C). The gene *cry* (cryptochrome) is a major photoreceptor for circadian rhythms and its loss has been shown to cause defects in photoentrainment [31]. Meanwhile, the gene *Nca* (Neurocalcin) encodes a calcium-binding protein that is essential for promoting night-time sleep and inhibiting nocturnal hyperactivity [32]. The loss of these genes indicates that the bat fly may have lost normal circadian rhythms. In addition, the gene *Lk* (Leucokinin) and its receptor *Lkr* (Leucokinin receptor) together are involved in feeding control. The *Lk* and *Lkr* mutant fly exhibited reduced metabolic rate and locomotor activity, reduced diuresis, as well as increased resistance to starvation and desiccation [33]. This is particularly relevant for the bat fly, which specializes in blood feeding—a challenging dietary habit due to the high fluid content (∼78%) and relatively low caloric value of blood [34]. The loss of the aforementioned genes may have led to alterations in the normal circadian rhythm and metabolic adaptation of bat flies, potentially as an adaptive response to meet the unique energy metabolic requirements necessitated by their hematophagous lifestyle.

### Genes related to chemosensory receptors

Multiple families of chemosensory genes [i.e. odorant receptors (ORs), ionotropic receptors (IRs), gustatory receptors (GRs), odorant-binding proteins (OBPs), chemosensory proteins (CSPs) and sensory neuron membrane proteins (SNMPs)] play important roles in detecting odors, permitting insects to locate hosts and mating partners, and avoiding predators [35]. All sensory proteins identified in the bat fly accounted for <10% of those in *D. melanogaster* (CSP: 3; OBP: 6; GR: 5; OR: 1; IR: 7; SNMPs: 2) (Fig. 2e and Table S2E). In invertebrates, the conservation of CSPs and SNMPs is relatively stable, while variations primarily occur in the numbers of OBPs, GRs ORs and IRs. Differences in the number of chemosensory receptors were correlated with the diet structure and host range of the bat fly.

### Viral infection of bat flies by artificial inoculation or feeding on viremic mice

*Nodamura* virus (NoV) is the first isolated member of the *Nodaviridae* and is unique in its ability to lethally infect both mammals and insects [36]. Our previous studies have shown that NoV wild-type (WT), NoVΔB2 (containing three point-mutations in the RNA1 of NoV to terminate B2 expression) and NoVmB2 (expressing a mutant B2 protein defective in dsRNA binding) can infect mice and ticks, triggering antiviral RNAi responses [37–41]. We identified the B2 protein of NoV as a viral suppressor of RNAi (VSR) [37]. In this work, we extend this approach to microinject NoV into bat flies to assess virus replication and RNAi induction. Notably, a recent study has shown that viruses with high homology to NoV have been detected in viruses naturally carried by bats [42]. To verify whether the coronavirus can replicate in bat flies, we used recombinant SARS-CoV-2 GFP/delN virus-like particles (VLPs), in which the N gene of SARS-CoV-2 has been replaced by a reporter gene (green fluorescent protein, GFP) [43]. The recombinant virus can only complete its life cycle in cells expressing the viral N protein, allowing the recombinant virus to be tested for infection in a biosafety level 2 containment.

**Figure 3.**
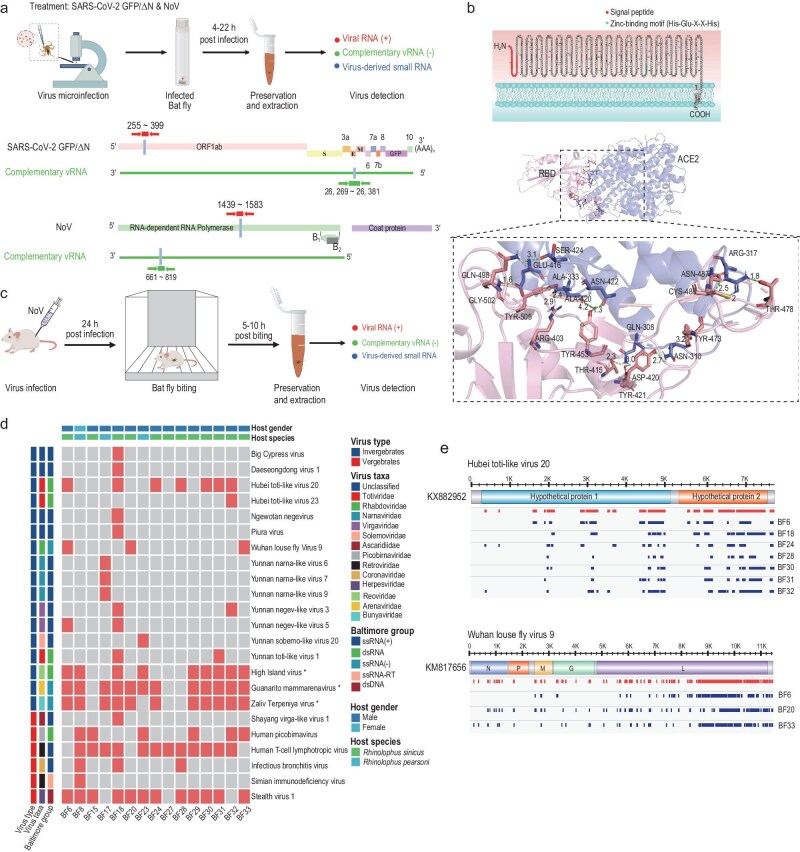
Virus infection and identification in the bat fly. (a) Schematic representation of microinjection and RNA sequencing in the bat fly. In brief, the process of microinjecting SARS-CoV-2 GFP/ΔN VLP and NoV into the leg of the bat fly, followed by the extraction of tissues for RNA sequencing analysis. Specifically, using sequences derived from the SARS-CoV-2 GFP/ΔN (255–399), we examined the presence of SARS-CoV-2 GFP/ΔN in bat flies to ascertain the success of the microinjection. Reverse primers were designed to amplify regions encompassing the E protein coding sequence (26269–26381), facilitating the detection of the E sequence as a marker for SARS-CoV-2 GFP/ΔN replication. A parallel approach was employed for NoV, with the utilization of distinct primers. (b) The secondary structure and topology of the bat fly ACE2 protein were predicted using Protter (http://wlab.ethz.ch/protter). The complex structure of the SARS-CoV-2 spike glycoprotein in conjunction with the bat fly ACE2. Interaction details at the Spike (RBD)–ACE2 interface are provided through PDBePISA prediction analysis. (c) Schematic presentation of the feeding of bat flies on NoV-infected mice, followed by tissue extraction from the bat flies for RNA sequencing. (d) The types of viruses detected in each bat fly sample and their classifications. Three viruses (High Island virus, Guanarito mammarenavirus and Zaliv Terpeniya virus), each with <10% genome coverage and marked with an asterisk, were identified in at least 9 out of 15 samples. (e) For Hubei toti-like virus 20 and Wuhan Louse Fly Virus 9, virus-assembled contigs were mapped to reference genome sequences. The raw number of viral contigs assembled for each sample and the pool of viral contigs obtained from all samples are labeled with different colors.

To evaluate the effectiveness of virus inoculation and its replication potential in bat flies, we analysed three types of viral RNAs in the specimens: (i) viral RNA, (ii) viral RNA complementary sequence (negative-sense viral RNA) and (iii) virus-derived small RNA (vsRNA) (Fig. 3a). Following microinjection of NoV into bat flies, we extracted total RNA a few hours post-inoculation. The detection of viral RNA in bat flies indicated successful inoculation (Fig. S3a and Tables S3A and S3B). While there was variability in the amount of viral RNA detected in NoV-inoculated bat flies, some specimens, particularly those inoculated for shorter durations, exhibited no detectable viral RNA complementary sequences (Table S3B). However, 20 hours after inoculation, viral RNA complementary sequences were identified in two bat flies, suggesting NoV replication in bat flies. Comparable results were observed in bat flies microinjected with SARS-CoV-2 GFP/delN VLPs. In these, viral RNA complementary sequences were identified after >20 hours of injection, though at lower levels than NoV, suggesting a limited replication ability of SARS-CoV-2 GFP/delN VLPs in bat flies (Fig. 3a, Fig. S3a and Table S3B). Additionally, the detection of a few negative-sense small RNAs from SARS-CoV-2 GFP/delN VLPs might signal fragments from the virus replication process (Fig. S3b).

The capability of viruses to infiltrate and replicate within bat fly cells may be influenced by the presence of specific cell surface receptors. Our access to the bat fly genome facilitates the investigation of these potential viral receptors. In humans, the primary receptor for the SARS-CoV-2 virus, which facilitates its entry into cells, is the Angiotensin Converting Enzyme II (ACE2) [44]. Our analysis reveals that the primary structure of the ACE2 protein is highly conserved among various species, including arthropod vectors, bats and humans, as depicted in Fig. S3c. Moreover, bioinformatics analysis of ACE2 protein sequences, focusing on the transmembrane helix and signal peptide, indicated that, while human and bat ACE2 proteins possess a carboxy-terminal transmembrane, none of the *D. melanogaster* ACE2 proteins has a membrane-anchoring sequence. Expanding our sequence analysis to encompass a broader range of species revealed that only the bat fly's ACE2 structure includes a transmembrane domain, similar to that in bats and humans (Fig. 3b and Fig. S3d). To explore the potential interaction between SARS-CoV-2 and the bat fly's ACE2, we simulated the structure of the SARS-CoV-2 S protein and the bat fly's ACE2 using the SWISS-MODEL online server, based on the established structure of the human ACE2 and SARS-CoV-2 receptor-binding domain (RBD) complex. This simulation of the potential protein complex structure unveiled that the SARS-CoV-2 S–ACE2 interface includes eight amino acid residues from ACE2 forming 14 hydrogen bond contacts with 12 residues from the SARS-CoV-2 RBD, as illustrated in Fig. 3b.

We had developed a virus–mouse–tick infection model to study the transmission and replication of the NoV [38]. To explore the host specificity of bat fly feeding, we established a virus–mouse–bat fly infection model (Fig. 3c). We inoculated 6- to 8-day-old suckling mice, chosen for their susceptibility to NoV-induced viremia and minimal hair and movement, facilitating observation of bat fly bites. Two days post-inoculation, we conducted a biting experiment with the infected mice and bat flies in a shared container. The bat flies quickly moved onto the mice, prompting a reflexive response from the mice when bitten (Video S1). In this experiment, a group of bat flies fed on NoV-infected mice for ∼10 hours. Post-feeding, we tested these bat flies for viral RNA and detected NoV RNA, confirming successful blood feeding. Additionally, the detection of negative-sense viral nucleic acid in the bat flies suggests potential NoV replication within the insects following blood feeding (Table S3B).

### Diversity of viruses in bat flies

During the viral replication, viral RNAs are susceptible to degradation by various antiviral mechanisms, including RNAi, RNA decay and the RNase L pathway. This degradation process leads to the accumulation of virus-derived small RNAs (vsRNAs) in infected cells [17]. Sequencing small RNA (sRNA) from these cells has been a successful strategy for virus discovery and characterization [45,46]. This approach, which relies on both homology-dependent and homology-independent methods, has been effectively utilized for over a decade [47,48].

To ascertain the diversity of virus present in bat flies, we selected 15 bat flies and performed sRNA sequencing on each bat fly sample, yielding between 4 781 034 and 19 204 671 reads (Tables S3C and S3D). Initially, the sRNA reads were aligned against known vertebrate and invertebrate virus reference sequences, subsequently assembling them into virus contigs. A portion of the total reads was successfully mapped to various viral genomes (0.7%–6.3% for vertebrate viruses; 0.5%–4.3% for invertebrate viruses) and a significant percentage to the bat fly genome (ranging from 11.3% to 94.7%). These sequences were then compared with known virus sequences using BLASTN to identify viral pathogens within the assembled contigs. The analysis identified viruses belonging to 13 families, alongside one unclassified viral taxon (Genus: Nelorpivirus), in the bat fly (Fig. 3d and Tables S3E–S3G). This included double-stranded DNA (dsDNA) viruses (Herpesviridae), positive single-stranded RNA [(+) ssRNA] viruses (Ascaridiidae, Coronaviridae, Narnaviridae, Retroviridae, Solemoviridae, Virgaviridae and Nelorpivirus), negative single-stranded RNA [(–) ssRNA] viruses (Arenaviridae, Bunyaviridae and Rhabdoviridae), single-stranded RNA-RT (ssRNA-RT) viruses (Retroviridae) and double-stranded RNA (dsRNA) viruses (Reoviridae and Totiviridae). Subsequent comparative analyses highlighted significant variances in the types and quantities of viruses across different bat fly samples. Significantly, sample BF18 displayed the greatest abundance of viral activity, evidenced by the detection of sequences from 15 unique viruses, whereas sample BF27 demonstrated minimal viral detection. Among the identified viruses, sequences closely resembling Wuhan Louse Fly Virus 9 were most frequently detected in samples BF6, BF20 and BF33 (Fig. 3d). These were assembled into 45, 47 and 44 contigs, respectively. Although most virus-assembled groups exhibited relatively short overlaps, their overall continuity was notably high, with consistencies ranging from 93.44% to 94.56% (Fig. 3e and Table S3F). Additionally, the invertebrate virus with the highest coverage depth identified was the Hubei toti-like virus 20 (Table S3F).

Benefitting from our preservation of certain bat tissues, we were able to concurrently analyse the virome of a specific bat fly alongside that of its corresponding bat tissue sample. We next performed VirusDetect analyses of sRNAs associated with muscle and liver of bats to investigate the virus–host dynamics. We compared the viral abundance of the seven viruses identified in the bat fly BF6 sample across the three data sets (BF6, muscle and liver of bat) by analysing the sRNA data (Fig. S3e). In the investigation of Hubei toti-like virus 20 and Wuhan Louse Fly Virus 9, substantial amounts of vsRNAs were observed within bat flies. In contrast, the concentration of this vsRNA was considerably lower in the liver and muscle tissues of the host bats. Steal virus 1 and Yunnan vegev-like virus 5 exhibited traits akin to the aforementioned two viruses. Conversely, Guanarito mammarenavirus and Zaliv Terpeniya virus demonstrated a distinct pattern. These two viruses produced abundant vsRNAs in bat tissues but their level of presence in bat flies was relatively minimal. The observed pattern suggests that there might be an enhanced propensity for infection and a somewhat increased representation within bats for both Guanarito mammarenavirus and Zaliv Terpeniya virus. Of particular note is High Island virus, which represented a unique scenario. Elevated levels of vsRNA were detected in both the bat flies and the bat tissues, indicating that High Island virus might have the capability to effectively infect both host species. Furthermore, we undertook an analysis of macrogenomic data from bat fly and bat samples, sourced from publicly available databases. Through meta-transcriptomic analysis of bat fly samples, sequences of the High Island virus were detected [5]. Complementing this, High Island virus sequences were also identified in samples derived from bat throat swabs and liver tissues (Table S3H) [49]. These findings imply that the High Island virus is widespread within bat populations and bat flies may play a significant role in its transmission.

### Virus infection can induce antiviral RNAi response in bat flies

**Figure 4.**
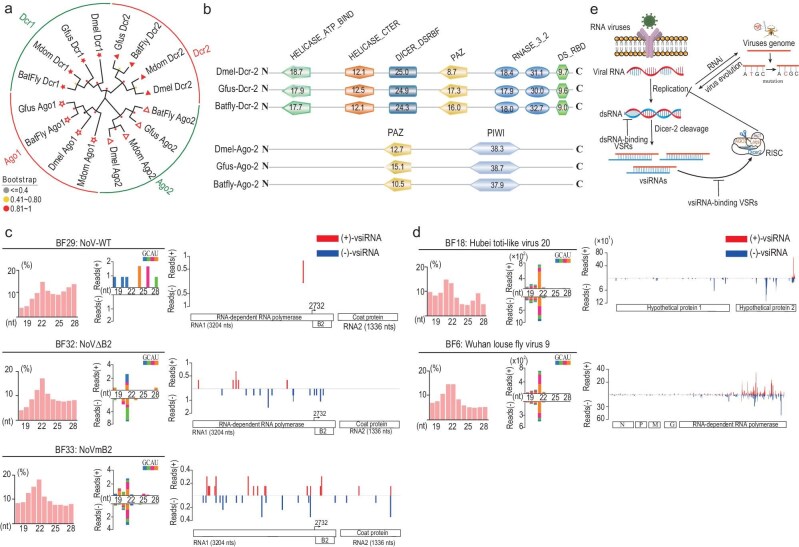
RNAi as an antiviral defense mechanism in the bat fly. (a) Phylogenetic analysis of Dicer/Argonaute proteins. Maximum likelihood phylogenetic tree based on the amino acid sequences of Dicer and Argonaute proteins. The color-coding of the labels indicates the approximate bootstrap values for each node. (b) The domain architecture of Dicer and Argonaute proteins is depicted, with domain scores provided by ScanProsite searches against the PROSITE protein domain database. (c) Analysis of vsiRNAs is conducted in the context of NoV WT, NoV-ΔB2 and NoV-mB2 infections. The properties of total small RNA reads, the size distribution of 18- to 28-nt vsiRNAs and the genomic coverage depth at each nucleotide position by 21- to 23-nt vsiRNAs are shown. Read counts are normalized as per million total reads. (d) Analysis of Hubei toti-like virus 20 and Wuhan Louse Fly Virus 9 vsiRNAs. The vsiRNAs associated with Hubei toti-like virus 20 and Wuhan Louse Fly Virus 9 are presented (refer to Fig. S4 for additional details). The analysis methodology is consistent with that described in Fig. 4c. (e) A distinct mechanism of viral evolution in response to RNAi.

We demonstrated that certain immune-related genes were absent in bat flies. To ascertain whether the antiviral RNAi pathway is effectively activated, we initially utilized the RNAi core genes of *D. melanogaster* and *G. morsitans* as references. Employing the reciprocal BLAST method, we identified homologous genes in the bat fly genome. With the exception of *AGO3*, five key genes were identified. *DICER1* and *AGO1* are implicated in the microRNA (miRNA) pathway, whereas *DICER2* and *AGO2* are integral to the siRNA pathway [16]. Subsequent phylogenetic analysis of proteins associated with these pathways across the three species revealed that identical genes from different species clustered together—a finding strongly corroborated by bootstrapping values (Fig. 4a). Our bioinformatics investigation confirmed the presence of all essential functional motifs in the identified Argonaute (Ago) proteins (PAZ: double-stranded RNA binding, PIWI: RNase activity execution), Dicer (DCR) enzymes (including N-terminal helicases, DCR-dsRNA Binding Fold, PAZ domain, two C-terminal Ribonuclease III domains and dsRNA-binding domain) and Drosha (C-terminal Ribonuclease III domain and the dsRBD) (Fig. 4b).

Subsequently, we microinjected NoV WT, NoVΔB2 and NoVmB2 viruses into bat flies, respectively. Post-inoculation, the presence of NoV RNA in the bat flies was confirmed, validating the success of the inoculation process. Analysis of sRNA sequencing data revealed the presence of virus-derived small RNAs, predominantly 21 nucleotides (nts) in length, in bat flies infected with NoVΔB2 and NoVmB2 (Fig. 4c). These small RNAs displayed characteristics akin to those observed in fruit flies and mosquitoes infected by viruses, suggesting the induction of an antiviral RNAi response in bat flies [16,17]. The origin of these small RNAs can be traced to the cleavage products of virus replication intermediates, further indicating active replication of NoV in the bat flies [37]. In contrast, in bat flies inoculated with NoV WT, we observed a subset of virus-derived small RNAs that lacked the signature cleavage patterns of Dicer2 (Fig. 4c). This observation may imply that the B2 protein in NoV WT can effectively inhibit the antiviral RNAi pathway in bat flies.

To clarify whether the viruses inherently carried by bat flies trigger the antiviral RNAi pathway, we conducted an analysis focusing on the Hubei toti-like virus 20 and Wuhan Louse Fly Virus 9, which are more prevalent in bat flies. We observed a significant presence of virus-derived small RNAs from both Hubei toti-like virus 20 and Wuhan Louse Fly Virus 9 in samples from BF6 and BF18, respectively (Fig. 4d and Fig. S4). These small RNAs have a peak length of 21 nt and were uniformly distributed across the viral gene sequence, with enrichment observed towards the end of the viral genome. These findings imply that the antiviral RNAi response may be a widespread mechanism in bat flies.

## DISCUSSION

Dipterans represent a diverse and ecologically significant group of insects, encompassing vectors of both human and animal diseases, including species such as mosquitoes, black flies and tsetse flies [50–52]. The sequencing and comparative analysis of dipteran genomes have yielded insights into their evolutionary relationships, adaptations and gene functions. Within the Hippoboscidae superfamily (Diptera), notable families include Glossinidae (tsetse flies), Hippoboscidae (bird flies and ked flies), Nycteribiidae and Streblidae [6]. One dipteran species of particular interest is the tsetse fly, known for its role as a vector of African trypanosomes, which are responsible for causing sleeping sickness in humans. The genome of the tsetse fly had been sequenced and annotated, revealing a genome size of 366 Mb and encompassing 12 308 protein-coding genes [50]. Within the Nycteribiidae family and its closest relatives in the Streblidae family, the species, commonly referred to as ‘bat flies’, have evolved to become obligate parasites, adapted to feeding on the blood of bats [7]. Notably, the genome size of the *Phthiridium* sp. we identified is less than half that of the tsetse fly. The evolution of parasitism has led to significant reductions in genome size and gene numbers [53]. The diminished genome size of the bat fly may be associated with its specialized parasitic lifestyle. For example, the Toll/Imd signaling pathways are responsible for the immune response to fungi and most bacteria, whether gram-positive or gram-negative [21]. Given the distinct species composition and lower bacterial abundance in blood, the genes in the Toll/Imd signaling pathway may accumulate deleterious mutations over extended evolutionary periods due to relaxed selection pressures, potentially leading to their eventual loss.

Xu *et al*. recently evaluated five types of blood-sucking parasitic arthropods on the surface of bats in Yunnan, China and identified a variety of viruses including 14 different virus families or orders. They also found three potential arboviruses, two of which belonged to Rhabdoviridae (Yunnan rhabdo-like virus 5 and Wuhan Louse Fly Virus 11) [5]. Identifying the exact viruses in a single ectoparasite is difficult using meta-transcriptomics because of the low amount of nucleic acid available. Xu *et al.* employed a strategy in which several individual specimens were pooled for sequencing. However, this strategy could not distinguish the specific viral species in each ectoparasite. In this study, we conducted sRNA sequencing to analyse the diversity of viruses carried by individual bat flies. From 15 samples, we were able to distinguish viruses originating from at least 13 different viral families. Notably, one bat fly (BF18) was found to harbor sequences from as many as 15 distinct virus species. In our analysis, viruses with relatively high abundance, such as toti-like virus and Wuhan Louse Fly Virus, were also identified in a previous study [5]. Intriguingly, we found a few gamma coronaviruses [infectious bronchitis virus (IBV)] sequences in three bat flies. IBV typically infects birds and there have been no reports of bats carrying this virus [54]. However, it is worth noting that a study suggested that cave bats (*Myotis lucifugus*) can exhibit symptoms or death when inoculated with the IBV through various infection routes [55]. Given the limited IBV sequences obtained in our analysis, further study is needed to confirm this finding. More recently, Zhang and colleagues reported a comprehensive and systematic study on the diversity, ecology, evolution and transmission of viruses in small mammals such as rodents, bats and shrews. Notably, they found a large number of viruses that were previously thought to be invertebrate-specific or related. Some of these viruses, discovered for the first time in mammals, are distributed in multiple organs and are highly abundant. These results suggest that these arthropod-specific or related viruses infect small mammalian hosts and do not originate solely from the host's feeding [56]. In this work, using sRNA sequencing, we simultaneously determined the viral diversities in a specific bat and its parasitic bat fly. A previous study reported that High Island virus—a reovirus isolated from mosquitoes [57]—had a high prevalence in our bat fly samples. We also detected abundant sequences of High Island virus in the muscle and liver tissues of the bat, suggesting that this reovirus could be an arbovirus for bats.

In invertebrates, OBPs predominantly function to bind odor molecules specifically and facilitate their transport. In contrast, ORs play a central role in chemical sensing, being crucial determinants of the sensitivity and specificity of olfactory perception [58]. A reduction in the number of OBPs and ORs may suggest a more simplified odor transport mechanism in bat flies, with a limited range of recognizable odor molecules. GRs are generally involved in detecting non-volatile substances linked to sweet tastes. Their reduction in bat flies aligns with their specialization in blood feeding, mirroring observations in Glossina species [50]. On the one hand, the diverse adaptive characteristics of bat flies imply their potential physiological and morphological aptitude for parasitizing bats within cave environments. On the other hand, our findings demonstrate that bat flies engage in the act of biting other mammalian species, raising the possibility that they may incidentally bite companion animals and even humans, thereby potentially enabling the cross-species transmission of pathogens. Moreover, blood is generally considered to be rich in diverse nutrients including amino acids, vitamins and other metabolites, which may be a compensatory mechanism for the parasitism and symbiosis such as fungal parasites and cicadas [59].

Kemenesi *et al.* recently identified and characterized the Lloviu virus (LLOV)—a filovirus family member that includes Ebola and Marburg viruses. Their research highlights the potential role of bat-associated parasites, such as bat flies, in the natural ecology of LLOV, suggesting that LLOV could be a vector-borne pathogen for bats with possible spillover to other mammals, including humans [11]. Hypothetically, arthropod ectoparasites might carry coronaviruses by biting infected hosts, harboring the virus on their mouthparts, exoskeleton or internally through ACE and integrin binding [4]. The virus could then be transmitted to other hosts through regurgitated blood during subsequent feedings. Previous reports claimed that cells of arthropods such as mosquitoes do not support the replication of SARS-CoV-2, but these results did not verify the potential infection of bat flies, which are most likely to contact various coronaviruses by sucking viremic bats in nature [60]. Upon inoculating bat flies with SARS-CoV-2 GFP/delN VLPs via microinjection, only negligible viral replication was observed, suggesting that SARS-CoV-2 GFP/delN VLPs may not replicate effectively in bat flies. Nonetheless, the potential of bat flies as mechanical vectors in coronavirus transmission should not be dismissed and warrants further investigation.

A previous study showed that a circadian-clock-controlled immune system might allow an organism to anticipate daily changes in feeding and the associated risk of infection or tissue damage to the host [61]. Disruption of circadian rhythms has been linked to inflammatory pathologies and metabolic disorders. The loss of genes related to circadian rhythms and Toll/IMD pathways in bat flies suggests the intrinsic interaction between circadian rhythm, metabolic adaptation and immune response. Circadian rhythm participates in metabolic process and regulates the functions of immune responses. Moreover, it is well established that activating immune response cascades (e.g. Toll and IMD pathways) requires a metabolic switch to meet the demand of the energy surge. Therefore, the loss of these genes suggests that infections by viruses may not activate such immune response cascades in bat flies. Instead, the RNAi may be the predominant antiviral response in this species.

Virus and host co-evolve, generating genetic variation that affects their fitness, virulence and transmission. These virus–host interactions are shaped by various factors, including environmental conditions, diversity and specificity of the host, viral diversity and recombination, and concurrent infections with other pathogens [15]. Gaining insights into these evolving interactions is essential for understanding the molecular basis of viral diseases. Studies have shown that exo-siRNA-mediated targeting of the West Nile virus (WNV) genome in mosquitoes increases the mutational diversity and fitness of WNV populations [62,63]. Similarly, Grubaugh *et al.* also reported that genetic bottlenecks, RNAi-mediated diversification and selective constraints jointly drive Powassan virus evolution in ticks [64]. These observations indicate that the RNAi pathway, due to its effectiveness and sequence specificity, may significantly influence the rate and manner of viral evolution. In this study, we inoculated bat flies with NoV to demonstrate the role of the RNAi pathway within these flies for the first time. When NoV∆B2 and mB2, both lacking suppressor function, were injected into the bat flies, the RNAi pathway was induced, producing Dicer2-specific cleavage products. In contrast, when inoculated with the NoV WT containing the suppressor, we did not detect canonical Dicer2 cleavage products, indicating that the suppressor protein B2 can inhibit the RNAi response in bat flies. Concurrently, we identified abundent 21-nt siRNAs from the Hubei toti-like virus 20 and Wuhan Louse Fly Virus 9 carried by the bat flies, suggesting that these viruses induce an RNAi pathway response during replication. As a control, although we detected a large number of sense and antisense sRNAs from High Island virus in the bat flies, these randomly produced sRNAs lacked Dicer2 cleavage characteristics, potentially indicating that High Island virus encodes a suppressor protein to antagonize the RNAi pathway. Given that bat flies carry various types of viruses, it is likely that their RNAi pathways play a significant role in the diversity and evolution of these viruses (Fig. 4e) given that bat flies reproduce via viviparous puparity, rather than oviparity like mosquitoes and ticks. Thus, it is challenging to establish axenic breeding of bat flies and compare additional pathways exhibiting significant expression changes potentially involved in antiviral defense in bat flies.

Environmental factors such as temperature, humidity and light may influence the development and behavior of bat flies [6]. Conducting thorough scientific studies on these non-model organisms poses substantial challenges, particularly in creating controlled experimental settings. In this work, we successfully infected bat flies by feeding them viremic mice and artificial inoculation. However, the limited replicative capacity of the recombinant SARS-CoV-2 used for inoculation via microinjection leaves uncertainties regarding infection via natural bat fly bites on coronavirus-infected bats. Further investigation is needed, involving the collection and examination of bat flies from coronavirus-infected bats. Alternatively, by utilizing our developed mouse–bat fly model, the potential for coronavirus replication in bat flies can be further explored. Blood-feeding arthropods, known to transmit various viruses including arboviruses such as dengue virus, may also harbor newly discovered invertebrate-specific or related viruses as potential arboviruses [56]. The sRNA analysis method employed in our research enables the concurrent assessment of viral diversity in mammalian hosts and their ectoparasites, aiding in elucidating virus–host dynamics. Future studies are needed to test the antiviral effect of bat flies from different geographical locations. Overall, our study presents a new genome catalog and reveals the evolutionary mechanisms underlying the parasitism of the bat fly, providing novel insights into the prevention of bat-borne zoonoses.

## MATERIALS AND METHODS

Animal care and experiments were conducted according to the guidelines established by the Regulations for the Administration of Affairs Concerning Experimental Animals (Ministry of Science and Technology, China, 2017). All procedures were also conducted following the approval of the Animal Experiment Ethics Committee in the Institution of Zoology, Chinese Academy of Sciences, China.

Detailed materials and methods are available in the Supplementary Data.

## Supplementary Material

nwae245_Supplemental_Files

## Data Availability

Genome and small RNA sequencing data of the bat fly have been deposited in the NCBI Sequence Read Archive under accession number PRJNA1039583 and PRJNA1041098, and are publicly available as of the date of publication.
