# Supplementary material for: Adaptive evolution of traits for parasitism and pathogen transmission potential in bat flies
Source: Natl Sci Rev. 2024 Jul 16;12(3):nwae245. doi: 10.1093/nsr/nwae245 (PMC11925017; doi:10.1093/nsr/nwae245)
Supplement: nwae245_Supplemental_Files [file nwae245_supplemental_files.zip › Supplementary_data.docx]

**Supplementary data for**

**Adaptive Evolution of Traits for Parasitism and Pathogen Transmission Potential in Bat Flies**

Materials and Methods

Figures. S1 to S4

Tables. S1 to S3

Video S1

**Materials and Methods**

**Bats and Bat flies**

Fifty-five bats (50 *Rhinolophus sinicus* and 5 *R*. *pearsoni*) were captured in a culvert located in Sangzhi County, Zhangjiajie City, Hunan Province, China. The captured bats were securely transported in sterilized cloth bags to a proximal temporary laboratory. In this laboratory, we collected bat flies from each bat, resulting in a total capture of 43 bat flies. Following the isolation of bat flies, a subset of the bats was released randomly, while others were subjected to dissection for the collection of tissues such as muscle and liver, which were earmarked for subsequent experimental analyses.

**Mice**

Kunming (KM) mice were procured from Spelford Biotechnology Co., Ltd., Beijing, China. For the virus transmission assay, we utilized eight-day-old suckling KM mice.

**Viruses**

The Nodamura virus (NoV) and its two variants, NoVmB2 and NoVΔB2, have been previously characterized [1]. SARS-CoV-2 GFP/delN virus-like particles (SARS-CoV-2 GFP/delN trVLP) were generously provided by Pro. Qiang Ding from Tsinghua University.

**Samples collection and nucleic acid extraction**

Specimens of bat flies were collected from *R*. *sinicus* and *R*.*pearsoni* utilizing blunt-end forceps. A portion of these bat flies were preserved in absolute ethanol or conserved in TRIzol reagent. Concurrently, select tissues from the captured bats were harvested and stored in TRIzol reagent for preservation. Genomic DNA from these samples was extracted employing the QIAGEN Genomic kit, while total RNA was extracted by TRIzol reagent used for further analysis.

**Genome sequencing and quality control**

A combination of the Oxford Nanopore Technologies (ONT), Illumina, and Hi-C sequencing was used to generate a high-quality genome assembly. ONT library was sequenced on the PromethION 48 platform. The nanopore raw reads were first transformed to base sequence in fastq format using Guppy (version: 3.2.2+9fe0a78) [2]. Any reads with a mean_qscore_template < 7 were then filtered, yielding a total of 64.03 Gb subreads (Table S1B). In addition, a paired-end (PE) library with a 350 bp insert size used for whole genome sequencing (WGS) and RNA-seq was constructed following the respective manufacturer’s instructions, and sequencing was performed on the MGISEQ-2000 platform with a strategy of 2 × 150 bp. Following adaptor sequences and low-quality reads were removed using fastp [3] (version 0.21.0) with default parameters, a total of 25.14 Gb of clean data were generated for subsequent analysis (Table S1B). To construct the chromosome-level genome assembly, the tissue samples were first performed cross-linked with formaldehyde for Hi-C sequencing. The restriction enzyme *DnpⅡ* was then added to digest the chromatin into units, marked by incubating with biotin-14-dCTP and ligated the units by biotinylation. The ligated DNA was finally sheared into 300-600 bp fragments and was also sequenced on the MGISEQ-2000 platform with a strategy of 2 × 150 bp, which generated 43.10 Gb data (Table S1B).

**Mitogenome assembly, annotation and taxonomic identification**

The mitogenome of this bat fly was also assembled based on the Illumina clean reads by MITObim [4] (version 1.9.1) with the species of Nycteribiidae as reference, including *Basilia ansifera*, *Nycteribia parvula*, *Dipseliopoda setosa* and *Phthiridium szechuanum*, and *Phthiridium* sp. (A GC-2023a isolate CHB10d) (Table S1C). The mitogenome annotation was performed with MITOS [5] (version 2).

The complete mitochondrial cytochrome c oxidase I (*COX1*) and cytochrome b (*Cytb*) genes were used to investigate the phylogenetic relationship of this species. The *COX1* and *Cytb* sequences of putative-related species (Table S1C) were retrieved from the “nucleotide” database of the National Center for Biotechnology Information (NCBI). The multiple sequence alignment was performed with MACSE version 2.06 [6]. Then, the RAxML [7] (version 8.2.12) was used to infer the phylogenetic tree with the maximum likelihood (ML) method. Higher than 99% similarity (99.15%-99.29%) in *COX1* and *Cytb* respectively were shown between the species of this study and *Phthiridium* sp. (A GC-2023a isolate CHB10d) (Figure S1b). The best quality of mitogenome assembly was obtained with the *Phthiridium* sp. (A GC-2023a isolate CHB10d) as a reference, further suggesting the assignment of *Phthiridium* sp.

**Whole genome assembly and annotation**

The k-mer analysis was first performed to evaluate the basic genomic characteristics of the bat fly by kmerFreq [8] with the *k* set as 17. Subsequently, the genome size was estimated using GCE [8] (version 1.0.2) software based on the k-mer frequency distribution.

To produce a de novo assembly, the ONT subreads were assembled into contigs using NextDenovo (version 2.3.1) [9]. The contigs were then polished with Racon [10] (version 1.3.1) using ONT long reads and NextPolish [11] (version 1.3.0) with Illumina short reads. Then, the Hi-C reads were mapped to the polished contig-level genome with bowtie2 (version 2.5.1) [12]. Valid interaction paired reads were identified and retained by HiC-Pro [13] (version 3.1.0) from unique mapped paired-end reads for further analysis. Finally, the contigs were anchored to chromosomes by LACHESIS [14].

The completeness of chromosome-level assembly was assessed using BUSCO5 [15] with the insecta_odb10 gene sets and CEGMA (version 2) [16]. To evaluate the accuracy of the assembly, all of the Illumina clean reads were mapped to the bat fly genome using BWA [17] (version 0.7.12-r1039); the mapping ratio, as well as genome coverage, was calculated with SAMtools [17] (version1.4), and the base accuracy of the assembly was assessed with BCFtools (version 1.8.0) [18]. Moreover, the Nanopore reads were also aligned back to the assembled genome using minimap2 version: r41) [19]. The mapping ratio, GC content, and average depth in each non-overlapping sliding window (50 kb) were then calculated. Besides, the genome-wide heterozygosity of the sequenced bat fly genome was calculated with reference to the method of Yang et al. [20].

Additionally, the collinearity of the protein-coding sequences of the bat fly and its related species *L. cuprina* using JCVI, and we visualized the collinearity region and detected the chromosome fusion and fission events using RectChr v1.36 (https://github.com/BGI-shenzhen/RectChr). The CIRCOS [21] (version 0.69-8) was used to visualize the chromosomes, GC content, gene, and repeat density, respectively.

**Genome annotation**

We initially identified repetitive sequences using different softwares. In brief, tandem repeats were predicted by Tandem Repeat Finder (version 4.07b) [22], and simple sequence repeats (SSR) were identified by GMATA (version 2.2) [23]. RepeatModeler [24] (version: open-1.0.11) was used to build the ab initio repeat library. RepeatMasker [25] (version: revision 1.331) was then applied against the ab initio repeat library and RepBase [26] (v20181026) library separately to search for homologous and novel repeats. In addition, we investigated the relationship between the genome size variation and transposons content among the bat fly and its related species (Table S1K).

Protein-coding genes were predicted in the repeats-masked genome by integrating de novo prediction, homology-based prediction, and transcripts-based prediction. Augustus [27] (version 3.3.1) was used to generate de novo prediction with internal gene models. The homology-based prediction was performed with GeMoMa [28] (version 1.6.1) based on published protein-coding sequences from *Aedes aegypti*, *Bactrocera dorsalis*, *D*. *melanogaster*, *G*. *morsitans*, *Paykullia maculata*, and *Stomoxys calcitrans* (Table S1J). RNA sequencing reads were aligned to the genome using STAR [29] (version 2.7.3a) and assembled by StringTie (version 1.3.4d) [30]. The open reading frames were then predicted using PASA (version 2.3.3) [31]. Finally, all the results derived from the above three methods were integrated into the final gene set using EvidenceModeler (version 1.1.1) [31].

The integrity of the final annotation was estimated using BUSCO5 with the insecta_odb10 gene sets. The gene functions of the gene set were annotated against the public databases, including SwissProt, NR, KEGG, KOG, and Gene Ontology with BLASTp (version 2.2.6) [32].

**Orthologous genes identification and phylogenetic analysis**

High-confidence “one-to-one” orthologous gene clusters were identified using the OrthoFinder pipeline [33] (version 2.5.5) with the protein-coding sequences among 13 species (*D*. *melanogaster*, *G*. *fuscipes*, *G*. *pallidipes*, *G*. *austeni*, *L*. *cuprina*, *L*.*sericata*, *M*. *domestica*, *Ceratitis capitata*, *B*. *dorsalis*, *Hermetia illucens*, *Anopheles stephensi*, and *A*. *aegypti*; Table S1K). The sequences were aligned using MACSE[6] (version 2.06) at the codon level. Poorly aligned regions with gaps and non-homologous fragments were removed using Gblocks [34] (version 0.91b) with strict parameters (“−t = c, −b5 = n”). Finally, high-quality multiple sequence alignments (MSAs) were obtained for subsequent analysis.

For phylogenetic tree construction, all MSAs were concatenated to one supergene and used for constructing a maximum likelihood (ML) phylogenetic tree by RAxML with 1000 bootstraps. Divergence time was estimated using MCMCtree from the PAML package [35] (version 4.9e), which combines with a molecular clock model. Three fossil-calibrated time points (Table S1M) referred to the TimeTree database [36].

**Gene family expansion and contraction**

Gene family expansion or contraction analyses were performed by Café [37] (version 4.2.1) based on the results from OrthoFinder. Gene families with a *P* value < 0.05 were considered as significance.

**Positive selection analysis**

For each orthologous group, we estimated the rates of nonsynonymous (*d*_N_) and synonymous (*d*_S_) (*ω* = *d*_N_/*d*_S_) in the CODEML program implemented in PAML [35] (version 4.9e) with the phylogenetic from this study. First, the branch-site model was used for detecting PSGs. Specifically, the bat fly lineage was labeled as the foreground branch and a likelihood ratio test (LRT) was conducted to examine whether the branch-site model containing positively selected codons (*ω* > 1) fits better than the null model, which only includes neutral selection (*ω* = 1) or negative selection (*ω* < 1). Using the Bayes Empirical Bayes (BEB) analysis, positively selected sites (PSSs) were estimated with posterior probabilities ≥ 0.80. Multiple testing correction was performed via a false discovery rate (FDR) < 0.05. To minimize the potential effects of false positives, we conducted a manual check the poorly conserved region, such as close to insertions or deletions or surrounded by large gaps.

**Functional enrichment analysis**

GO and KEGG enrichment analyses for expanded, contracted gene families, and PSGs were performed using Metascape (version 3.5.20230501) [38]. Meanwhile, the results of GO enrichment were filtered redundancy and visualized by REVIGO (version v1.8.1) [39]. Furthermore, we used literature searches and FlyBase [40] (version: FB2023_05) to explore the potential biological functions of each gene associated with the adaptive evolutionary trait of the bat fly.

**Identification of chemoreceptor, opsin, and RNAi genes**

We obtained all known chemoreceptor protein sequences related to *D*. *melanogaster* and *G*. *morsitans* by conducting exhaustive searches from previously published papers [41, 42]. First, we performed a homology search (E-value ≤ 1*10^-5^) using the BLASTp [32] (version 2.2.6) program against the bat fly genome to find candidate genes. Then, the hmmer [43] (version 3.0) with the default parameters setting to perform searches against the Pfam [44], a database of protein domain models to confirm the identity of the candidate genes. Hidden Markov models (HMMs) of CSP (PF02949), OBP (PF01395), IRs (PF00060), GRs (PF08395), ORs (PF02949), and SNMPs (PF01130) were used [45]. The candidate genes obtained by BLASTp containing the HMM profiles were regarded as confirmed genes. In addition, to confirm the presence of opsin and RNAi genes in the genome of the bat fly, we retrieved *Rh1-6*, *AGO*, *DCR*, and *Drosha* sequences from the VectorBase database (version: release 65) (https://vectorbase.org/vectorbase/app/) using annotated homologous gene sequences of the *D*. *melanogaster* and tsetse flies (*G*. *austeni*, *G*. *brevipalpis*, *G*. *fuscipes*, *G*. *morsitans*, and *G*. *palpalis*) as query sequences (BLASTp; E-value ≤ 1*10^-5^). BLAST hits with an identity to the query sequence lower than 40% were further confirmed based on reciprocal blast best hits. Considering the key role of RNAi in the antiviral immunity of invertebrates, we further analyzed the conservation of functional domains using the ScanProsite tool. ML phylogenetic analysis was performed by the GTRGAMMA model with 1000 bootstraps in RAxML[7].

**Infection of bat flies**

In our provisional research facility, we undertook the collection of bat flies from their bat hosts. The immobilization of these bat flies was achieved using adhesive tape affixed to 6 cm Petri dishes. The dorsal surface of each bat fly was adhered to the sticky side of the tape, effectively immobilizing them for further processing. Subsequent inoculations were performed under a stereomicroscope using a Nanoinject III apparatus. The inoculation needles, fashioned from nano-inject glass tubes, were finely tapered to a point using a HL-1000 needle puller supplied by Gairdner Technology Company, Wuhan, China. Our initial protocol, inspired by the methodology described in Xu *et al*., for tick injections, involved intrathoracic administration [46]. However, this approach resulted in the mortality of bat flies within approximately 5-10 minutes post-injection. Alternatively, we administered 10 nL of either NoV (with a concentration of 3.6x10^3^ genome RNA copies) or SARS-CoV-2 GFP/delN trVLP into the leg of each bat fly to mitigate additional harm. Post-inoculation, each bat fly was individually housed in a collection tube. To simulate the bat flies' native, dark, and humid habitat, we custom-cut foam strips, saturated them in distilled water, and placed them in the collection tubes to maintain a high humidity environment. These tubes were then stored in darkness at 24-28 °C. As per our experimental design, these bat fly samples were subsequently preserved in TRIzol Reagent for further analysis.

In the context of mice infection, six-day-old KM suckling mice were subjected to intraperitoneal injections with either NoV WT or NoV mB2. Each inoculum comprised a preparation containing 7x10^6^ copies of genomic RNA1 of NoV. At 48 hours post-infection with either NoV WT or NoV mB2, we facilitated the feeding of bat flies on these NoV-infected mice. Following this exposure, the bat flies were carefully harvested and preserved in TRIzol Reagent.

**Quantification of the virus by real-time qPCR (RT-qPCR)**

Total RNA was extracted from the tissue samples of the bat flies and the infected mice using TRIzol reagent following the manufacturer’s protocol. For the synthesis of complementary DNA (cDNA), 4 μL of the extracted total RNA was utilized, employing the HiScript® III RT SuperMix for qPCR. Subsequently, RT-qPCR was conducted using the ChamQ Universal SYBR qPCR Master Mix. This was performed with a 1:10 dilution of the synthesized cDNA. All procedures were executed in accordance with the manufacturer’s protocol. The specific primers employed for the RT-qPCR analysis are detailed in Table S3A.

**Determination of viral inoculation and replication by sequencing**

The cDNA was generated from 5μL total RNA using HiScript® III RT SuperMix. Based on the viral sequence, we designed specific primers to amplify segments of the virus. To circumvent the generation of false-positive signals during the PCR process, we subjected the amplified fragments to sequencing validation. Samples whose viral amplification sequences were in complete concordance with the designed primers were positive, indicating successful viral inoculation. Specifically, to confirm the presence of SARS-CoV-2 GFP/delN virus-like particles (VLP) (nucleotides 255-399) in bat flies and assess the efficacy of the injection, sequence-specific assays were implemented. Additionally, for the detection of NoV in bat flies and to validate the injection process, our initial assays focused on the region spanning nucleotides 1439-1583 of the NoV RNA1. The primers utilized for this analysis are comprehensively listed in Table S3A.

SARS-CoV-2 GFP/delN VLPs and NoV are both positive-sense RNA viruses that generate negative-sense viral RNA (minus-strand RNA) sequences during their replication process [47]. To ascertain whether replication of SARS-CoV-2 GFP/delN VLPs and NoV occurred, we engineered specific primers designed to exclusively detect the production of these negative-sense viral RNA sequences. Similarly, we conducted sequencing validation on the corresponding amplified fragments. The detection of the correct negative-sense viral RNA sequences serves as an indicator of viral replication. Specifically, we designed reverse primers targeting the minus-strand RNA of SARS-CoV-2 GFP/delN VLP (nucleotides 26238-26409) [47]. This approach resulted in amplification products that included the coding sequence of the envelope (E) protein. The detection of the E protein (nucleotides 26269-26381) was then carried out to confirm the replication of SARS-CoV-2 GFP/delN VLP in bat flies. For NoV, reverse primers were engineered to amplify a region following nucleotide 661 of the RNA polymerase coding gene. PCR analysis was subsequently performed to affirm NoV replication in the bat flies, concentrating on the region between nucleotides 661-819. The primers used in this analysis are listed in Table S3A.

**Virus identification by small RNA sequencing**

Many studies have established the efficacy of VirusDetect [48] (version 1.6) in the proficient detection of both known and novel viruses [49]. In this study, sequencing raw reads were trimmed for adapter sequence, and small RNA sequence sets were filtered to retain lengths of 18 to 35 nucleotides. We utilized purified small RNA sequences as the input for VirusDetect. This software was then employed for de novo assembly and contig mapping against a viral reference database, using parameters set as " coverage_cutoff = 1, depth_cutoff = 5." There were high-identity coverage and depth_cutoff for contigs, which we considered to be high-confidence virus detections. We also examined the frequency of occurrence of viruses from low-coverage viruses who only met criteria for depth_cutoff in different sample. If viruses were detected in more than half of the samples, we hold the belief that it is not merely a coincidence but rather a genuine presence. In addition, to mitigate the interference of host sequences, we implemented a filtration step using the bat fly genome, aiming to exclude endogenous small RNAs. Furthermore, by aligning sRNA reads to the complete genome of the newly identified virus, we engaged in the delineation of virus-derived siRNAs, a critical aspect for understanding viral-host interactions and viral replication mechanisms.

**Assembly of metagenome**

We acquired raw metagenomic sequencing data from various sources, including mixed bat fly samples (accession number: SRR1298412) from the NCBI Sequence Read Archive (SRA), mixed samples of bat liver tissues (accession number: SRR15616373), and mixed samples of bat throat swabs from the Genome Sequence Archive (GSA) at the National Genomics Data Center, China National Center for Bioinformation (accession number: CRA006652). Our aim was to utilize a metagenomic approach to investigate the pathogen distribution within these diverse biological samples. In our methodology, we first employed the fastp software to filter raw RNA sequencing reads, followed by the removal of reads corresponding to the host genome. Subsequent to this filtration process, we applied MEGAHIT [50] (version 1.1.3) for de novo assembly of the sequence reads into contigs. Only contigs with a minimum length of 500 nucleotides were retained for further analysis. These contigs were then subject to blast analysis (BLASTn; E-value ≤ 1*10^-5^) against viral sequences present in the GenBank database, thereby allowing for enhanced verification and identification of viral constituents within the samples.

**Spike RDB-ACE2 interface analysis**

Utilizing the available crystal structure of human ACE2 in complex with the SARS-CoV-2 spike (S) receptor-binding domain (RBD) (PDB: 6LZG), which is deposited in the Protein Data Bank (https://www.rcsb.org/), we simulated the structure of SARS-CoV-2 S and ACE2 derived from the bat fly. This simulation was conducted using the SWISS-MODEL online server [51]. Subsequently, the structures were docked employing the ZDOCK docking server v3.0.2 [52]. Furthermore, the binding energies of these docked complexes were computed using PDBePISA (<https://www.ebi.ac.uk/pdbe/pisa/>). The molecular interactions and conformations of the docked structures were visualized and analyzed using the PyMOL software (version 1.7.2.1) [53]. Additionally, the predicted membrane topology of the ACE2 protein was visualized using Protter (<http://wlab.ethz.ch/protter>).

**Deep sequencing and bioinformatic analysis of small RNAs**

Total RNA was used as input material for the RNA sample preparations. Small RNA libraries were constructed using NEB Next Multiplex Small RNA Library Prep Set for Illumina (NEB E7300L) following the manufacturer’s protocols. The qualified libraries were pooled and sequenced on Illumina sequencing with the SE50 strategy. Adapter sequences were removed from small RNA reads, which were scaled to reads per million of total reads (RPM), and clean reads were aligned to a reference genome to determine the size distribution of virus-derived small RNAs using bowtie (version 1.2.2) with one mismatch settings. The final data analysis was performed with an in-house Perl script as described previously [54]. Pairs of 22-nt vsiRNA read with 2-nt 3’ overhangs (-2 peak) were computed using a previously described algorithm. Size distribution, the 5’-nucleotide preference, and the mapping position of vsiRNAs (per million total 18-28 nt reads) along the viral sequences are displayed.

**Statistical analysis**

Statistical analyses were conducted utilizing GraphPad Prism software, version 9 for Windows. Quantification of viral RNA levels was achieved using the ΔCt method, with the β-actin mRNA of the bat fly serving as the internal reference. For the assessment of gene expression levels of the relevant virus via RT-qPCR, we employed the ΔΔCt method.

**References**

1. Li Y, Lu J, Han Y *et al.* RNA interference functions as an antiviral immunity mechanism in mammals. *Science*. 2013; **342**(6155): 231-234.

2. Wick RR, Judd LM, Holt KE. Performance of neural network basecalling tools for Oxford Nanopore sequencing. *Genome Biology*. 2019; **20**(1): 129-.

3. Chen S, Zhou Y, Chen Y *et al.* fastp: an ultra-fast all-in-one FASTQ preprocessor. *Bioinformatics*. 2018; **34**(17): i884-i890.

4. Christoph H, Lutz B, Bastien C. Reconstructing mitochondrial genomes directly from genomic next-generation sequencing reads—a baiting and iterative mapping approach. *Nucleic Acids Research*. 2013(13): e129-e129.

5. Bernt M, Donath A, Jühling F *et al.* MITOS: improved de novo metazoan mitochondrial genome annotation. *Molecular phylogenetics and evolution*. 2013; **69**(2): 313-319.

6. Ranwez V, Douzery EJ, Cambon C *et al.* MACSE v2: toolkit for the alignment of coding sequences accounting for frameshifts and stop codons. *Molecular biology and evolution*. 2018; **35**(10): 2582-2584.

7. Stamatakis A. RAxML version 8: a tool for phylogenetic analysis and post-analysis of large phylogenies. *Bioinformatics*. 2014; **30**(9): 1312-1313.

8. Liu B, Shi Y, Yuan J *et al.* Estimation of genomic characteristics by analyzing k-mer frequency in de novo genome projects. *arXiv preprint arXiv:13082012*. 2013.

9. Hu J, Wang Z, Sun Z *et al.* An efficient error correction and accurate assembly tool for noisy long reads. *bioRxiv*. 2023: 2023.2003. 2009.531669.

10. Vaser R, Sović I, Nagarajan N *et al.* Fast and accurate de novo genome assembly from long uncorrected reads. *Genome research*. 2017; **27**(5): 737-746.

11. Hu J, Fan J, Sun Z *et al.* NextPolish: a fast and efficient genome polishing tool for long-read assembly. *Bioinformatics*. 2020; **36**(7): 2253-2255.

12. Langmead B, Salzberg SL. Fast gapped-read alignment with Bowtie 2. *Nature methods*. 2012; **9**(4): 357-359.

13. Servant N, Varoquaux N, Lajoie BR *et al.* HiC-Pro: an optimized and flexible pipeline for Hi-C data processing. *Genome biology*. 2015; **16**(1): 1-11.

14. Burton JN, Adey A, Patwardhan RP *et al.* Chromosome-scale scaffolding of de novo genome assemblies based on chromatin interactions. *Nature biotechnology*. 2013; **31**(12): 1119-1125.

15. Simão FA, Waterhouse RM, Ioannidis P *et al.* BUSCO: assessing genome assembly and annotation completeness with single-copy orthologs. *Bioinformatics*. 2015; **31**(19): 3210-3212.

16. Parra G, Bradnam K, Korf I. CEGMA: a pipeline to accurately annotate core genes in eukaryotic genomes. *Bioinformatics*. 2007; **23**(9): 1061-1067.

17. Li H, Handsaker B, Wysoker A *et al.* The sequence alignment/map format and SAMtools. *bioinformatics*. 2009; **25**(16): 2078-2079.

18. Danecek P, Bonfield JK, Liddle J *et al.* Twelve years of SAMtools and BCFtools. *Gigascience*. 2021; **10**(2): giab008.

19. Li H. Minimap2: pairwise alignment for nucleotide sequences. *Bioinformatics*. 2018; **34**(18): 3094-3100.

20. Yang L, Wei F, Zhan X *et al.* Evolutionary conservation genomics reveals recent speciation and local adaptation in threatened takins. *Molecular Biology and Evolution*. 2022; **39**(6): msac111.

21. Krzywinski M, Schein J, Birol I *et al.* Circos: an information aesthetic for comparative genomics. *Genome research*. 2009; **19**(9): 1639-1645.

22. Benson G. Tandem repeats finder: a program to analyze DNA sequences. *Nucleic acids research*. 1999; **27**(2): 573-580.

23. Wang X, Wang L. GMATA: an integrated software package for genome-scale SSR mining, marker development and viewing. *Frontiers in plant science*. 2016; **7**: 1350.

24. Flynn JM, Hubley R, Goubert C *et al.* RepeatModeler2 for automated genomic discovery of transposable element families. *Proceedings of the National Academy of Sciences*. 2020; **117**(17): 9451-9457.

25. Chen N. Using Repeat Masker to identify repetitive elements in genomic sequences. *Current protocols in bioinformatics*. 2004; **5**(1): 4.10. 11-14.10. 14.

26. Jurka J, Kapitonov VV, Pavlicek A *et al.* Repbase Update, a database of eukaryotic repetitive elements. *Cytogenetic and genome research*. 2005; **110**(1-4): 462-467.

27. Stanke M, Keller O, Gunduz I *et al.* AUGUSTUS: ab initio prediction of alternative transcripts. *Nucleic acids research*. 2006; **34**(suppl_2): W435-W439.

28. Keilwagen J, Hartung F, Grau J. GeMoMa: homology-based gene prediction utilizing intron position conservation and RNA-seq data. *Gene prediction: Methods and protocols*. 2019: 161-177.

29. Dobin A, Davis CA, Schlesinger F *et al.* STAR: ultrafast universal RNA-seq aligner. *Bioinformatics*. 2013; **29**(1): 15-21.

30. Pertea M, Pertea GM, Antonescu CM *et al.* StringTie enables improved reconstruction of a transcriptome from RNA-seq reads. *Nature biotechnology*. 2015; **33**(3): 290-295.

31. Haas BJ, Salzberg SL, Zhu W *et al.* Automated eukaryotic gene structure annotation using EVidenceModeler and the Program to Assemble Spliced Alignments. *Genome biology*. 2008; **9**: 1-22.

32. Altschul SF, Gish W, Miller W *et al.* Basic local alignment search tool. *Journal of molecular biology*. 1990; **215**(3): 403-410.

33. Emms DM, Kelly S. OrthoFinder: phylogenetic orthology inference for comparative genomics. *Genome biology*. 2019; **20**: 1-14.

34. Castresana J. Selection of conserved blocks from multiple alignments for their use in phylogenetic analysis. *Molecular biology and evolution*. 2000; **17**(4): 540-552.

35. Yang Z. PAML 4: phylogenetic analysis by maximum likelihood. *Molecular biology and evolution*. 2007; **24**(8): 1586-1591.

36. Kumar S, Stecher G, Suleski M *et al.* TimeTree: a resource for timelines, timetrees, and divergence times. *Molecular biology and evolution*. 2017; **34**(7): 1812-1819.

37. De Bie T, Cristianini N, Demuth JP *et al.* CAFE: a computational tool for the study of gene family evolution. *Bioinformatics*. 2006; **22**(10): 1269-1271.

38. Zhou Y, Zhou B, Pache L *et al.* Metascape provides a biologist-oriented resource for the analysis of systems-level datasets. *Nature communications*. 2019; **10**(1): 1523.

39. Supek F, Bošnjak M, Škunca N *et al.* REVIGO summarizes and visualizes long lists of gene ontology terms. *PloS one*. 2011; **6**(7): e21800.

40. Gramates LS, Agapite J, Attrill H *et al.* FlyBase: a guided tour of highlighted features. *Genetics*. 2022; **220**(4): iyac035.

41. Robertson HM, Warr CG, Carlson JR. Molecular evolution of the insect chemoreceptor gene superfamily in Drosophila melanogaster. *Proceedings of the National Academy of Sciences*. 2003; **100**(suppl_2): 14537-14542.

42. Opondo O, Fredrick G. Genome-wide annotation of chemosensory and glutamate-gated receptors, and related genes in Glossina morsitans morsitans tsetse fly. University of the Western Cape, South Africa, 2014.

43. Potter SC, Luciani A, Eddy SR *et al.* HMMER web server: 2018 update. *Nucleic acids research*. 2018; **46**(W1): W200-W204.

44. Finn RD, Bateman A, Clements J *et al.* Pfam: the protein families database. *Nucleic acids research*. 2014; **42**(D1): D222-D230.

45. Li X, Li J-W, Sun W-X *et al.* Candidate chemosensory genes identified in the adult antennae of Sympiezomias velatus and binding property of odorant-binding protein 15. *Frontiers in Physiology*. 2022; **13**: 907667.

46. Xu Y, Zhong Z, Ren Y *et al.* Antiviral RNA interference in disease vector (Asian longhorned) ticks. *PLoS Pathogens*. 2021; **17**(12): e1010119.

47. Hogan CA, Huang C, Sahoo MK *et al.* Strand-specific reverse transcription PCR for detection of replicating SARS-CoV-2. *Emerging Infectious Diseases*. 2021; **27**(2): 632.

48. Zheng Y, Gao S, Padmanabhan C *et al.* VirusDetect: An automated pipeline for efficient virus discovery using deep sequencing of small RNAs. *Virology*. 2017; **500**: 130-138.

49. Xu C, Sun X, Taylor A *et al.* Diversity, distribution, and evolution of tomato viruses in China uncovered by small RNA sequencing. *Journal of Virology*. 2017; **91**(11): 10.1128/jvi. 00173-00117.

50. Li D, Luo R, Liu C-M *et al.* MEGAHIT v1. 0: a fast and scalable metagenome assembler driven by advanced methodologies and community practices. *Methods*. 2016; **102**: 3-11.

51. Biasini M, Bienert S, Waterhouse A *et al.* SWISS-MODEL: modelling protein tertiary and quaternary structure using evolutionary information. *Nucleic acids research*. 2014; **42**(W1): W252-W258.

52. Pierce BG, Wiehe K, Hwang H *et al.* ZDOCK server: interactive docking prediction of protein–protein complexes and symmetric multimers. *Bioinformatics*. 2014; **30**(12): 1771-1773.

53. DeLano WL. Pymol: An open-source molecular graphics tool. *CCP4 Newsl Protein Crystallogr*. 2002; **40**(1): 82-92.

54. Li Y, Basavappa M, Lu J *et al.* Induction and suppression of antiviral RNA interference by influenza A virus in mammalian cells. *Nature microbiology*. 2016; **2**(3): 1-9.

**
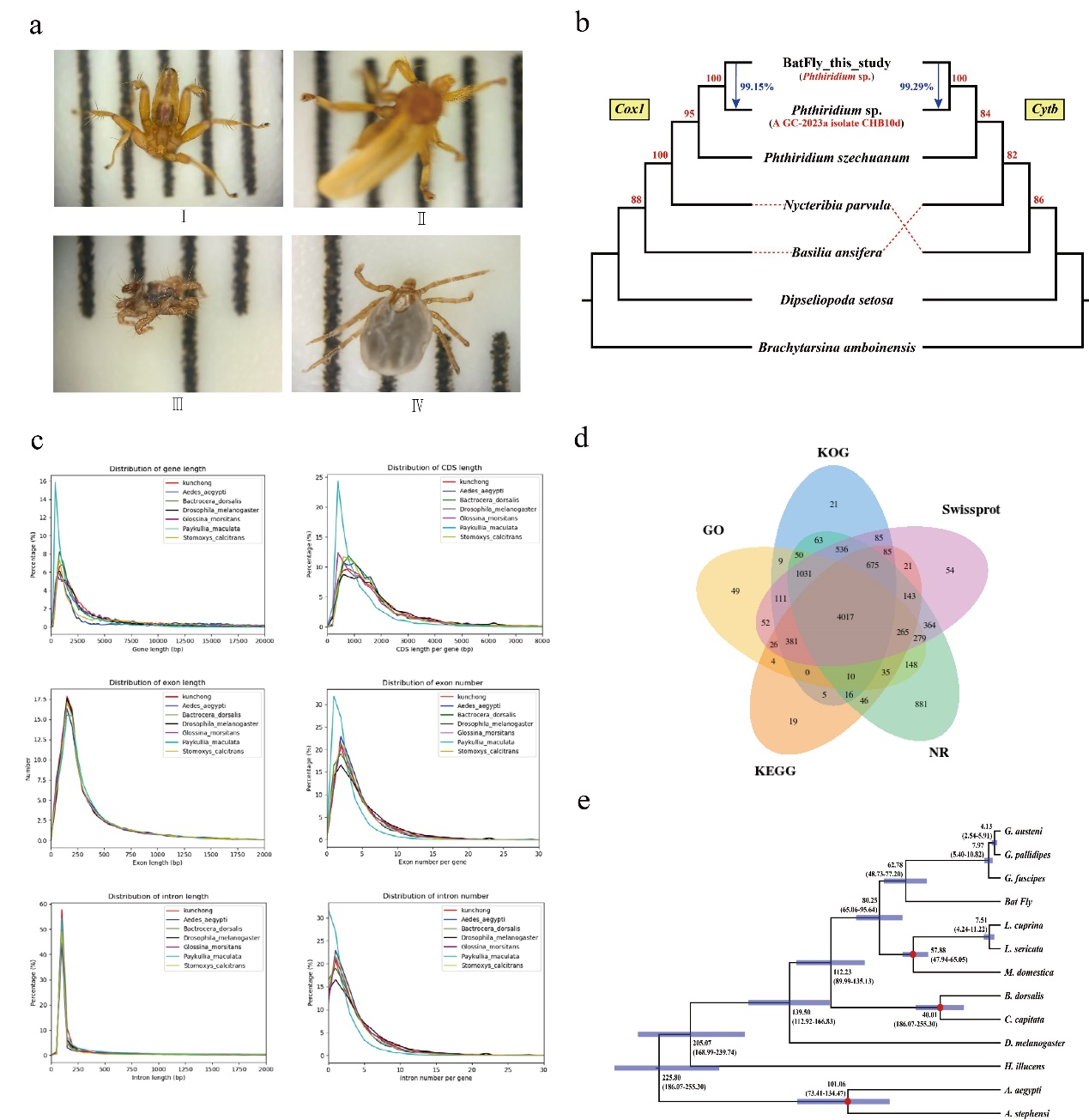
**

**Figure S1. Morphological and genomic characterization of the bat fly**

**a,** Bat surface parasites. Microscopic field images of parasites found on bat surfaces, including (Ⅰ) Nycteribiidae, (Ⅱ) Streblidae, (Ⅲ) mites, and (Ⅳ) ticks. The scale is represented with grid lines, each corresponding to one millimeter.

**b,** The bat fly sequenced in this study showed close taxonomic affinity to *Phthiridium* sp. (A GC-2023a isolate CHB10d). A maximum likelihood (ML) phylogenetic tree, constructed with RAxML and supported by 1000 bootstrap replicates, illustrates this relationship.

**c,** The distribution of gene length, coding sequence (CDS) length, exon length, intron length, and intron number in the bat fly genome is compared with related species. The bat fly is referred to as “kunchong” in this comparison.

**d,** A Venn diagram showcases the functional annotation of protein-coding genes in the bat fly genome, as identified through various databases including GO, KOG, SwissProt, KEGG, and NR.

**e,** The 95% confidence intervals of the phylogenetic tree featuring the bat fly and 12 other Diptera species. Three calibration points are marked with red circles, and purple bars surrounding each node indicate the 95% confidence intervals, which are detailed in parentheses.

**
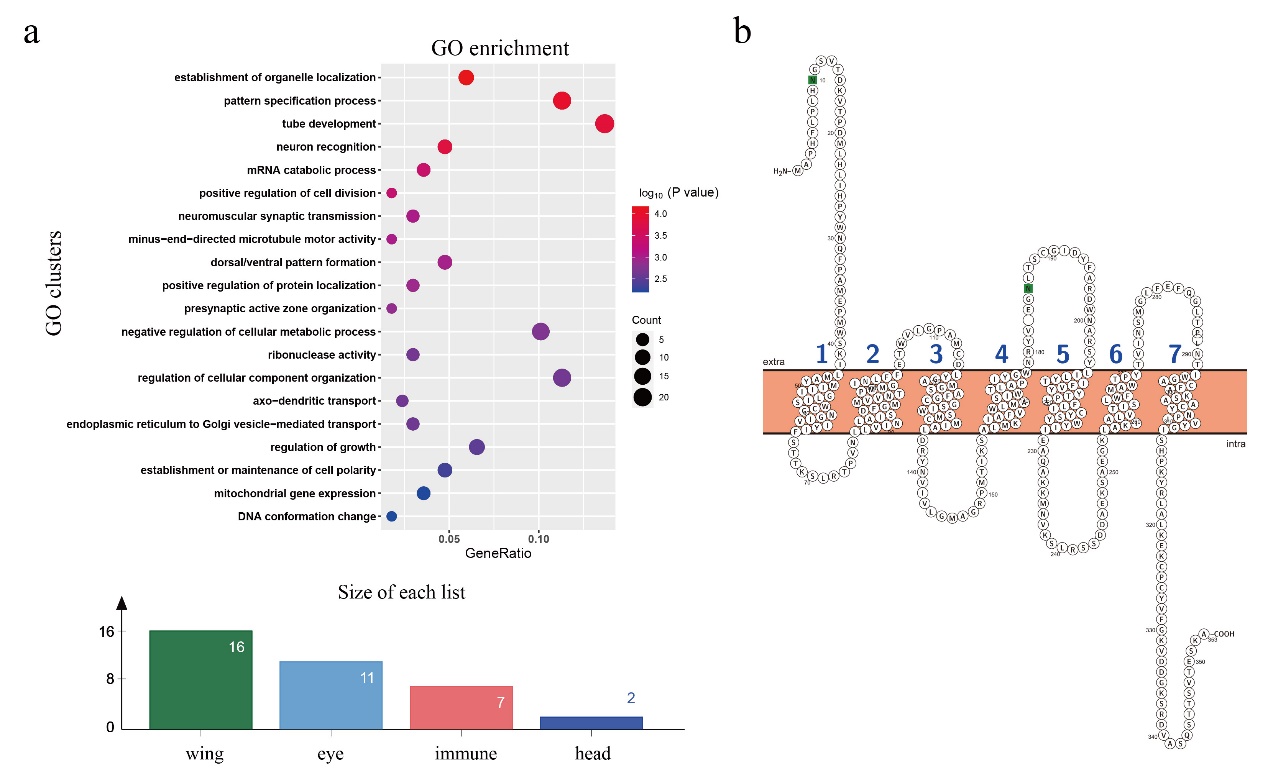
**

**Figure S2. Adaptive evolutionary signatures in bat fly genes**

**a,** Gene functional enrichment analysis of genes positively related to bat flies. The bubble chart displays the significance of enrichment (indicated by bubble color) and the gene count (reflected by bubble size). A histogram shows the number of genes under positive selection involved in morphological traits. Enriched Gene Ontology (GO) terms are highlighted, with a significance cutoff set at a P value < 0.05.

**b,** The schematic diagram represents the proposed secondary structure and topology of the Rhodopsin 1 (Rh1) protein in bat flies.

**
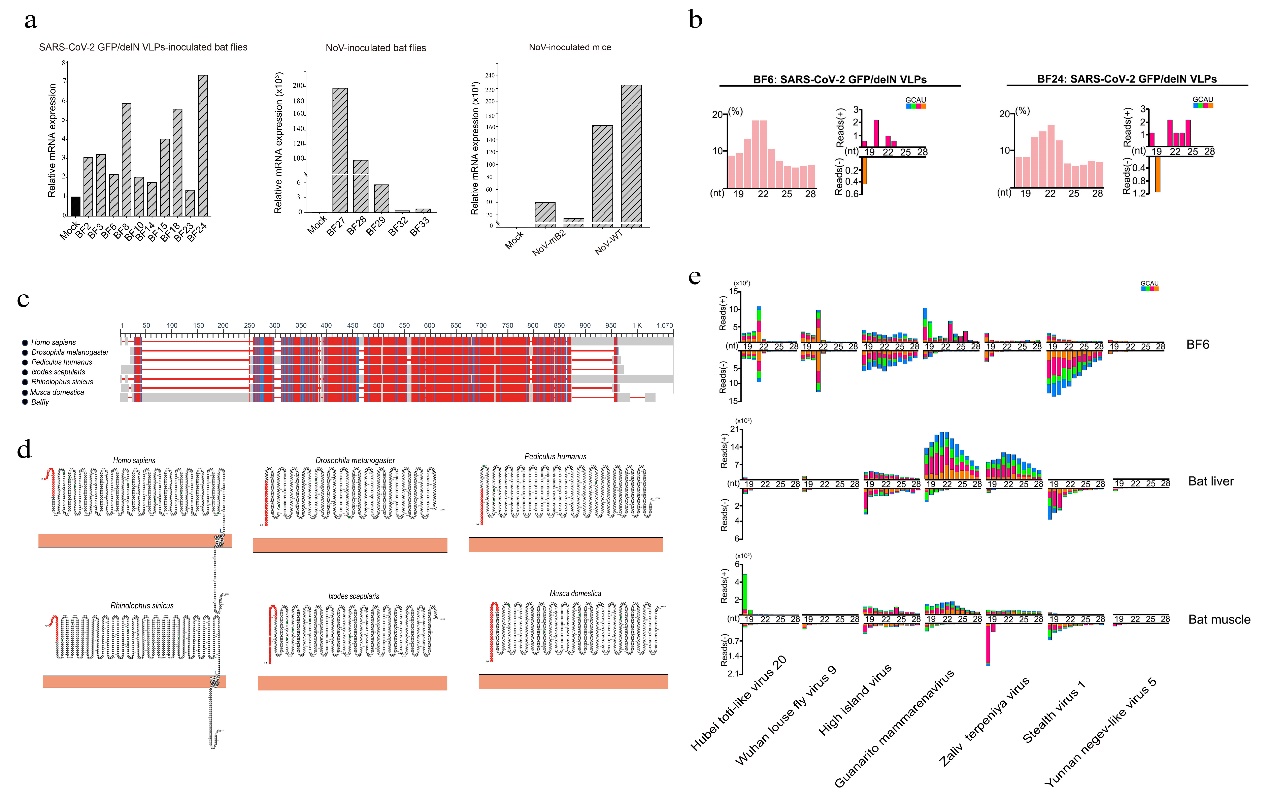
**

**Figure S3. Identification of viral RNAs and profiles of ACE2**

**a,** The determination of viral RNA levels through qRT-PCR across different samples.

**b,** Characterization of small RNAs was performed in the context of SARS-CoV-2 GFP/delN VLP infections.

**c,** Multiple amino acid sequence alignments for full-length sequences were conducted using COBALT, with parameters set to a Blast E-value of 0.005 and a maximum cluster distance of 0.8.

**d,** The schematic representation of the secondary structure and topology of the ACE2 protein from different species.

**e,** The viral abundance of the seven viruses identified in the BF6 samples. The abundance is compared across three datasets (BF6, and the muscle and liver tissues of bats), using sRNA data analysis, to understand the distribution and prevalence of these viruses in different biological samples.

**
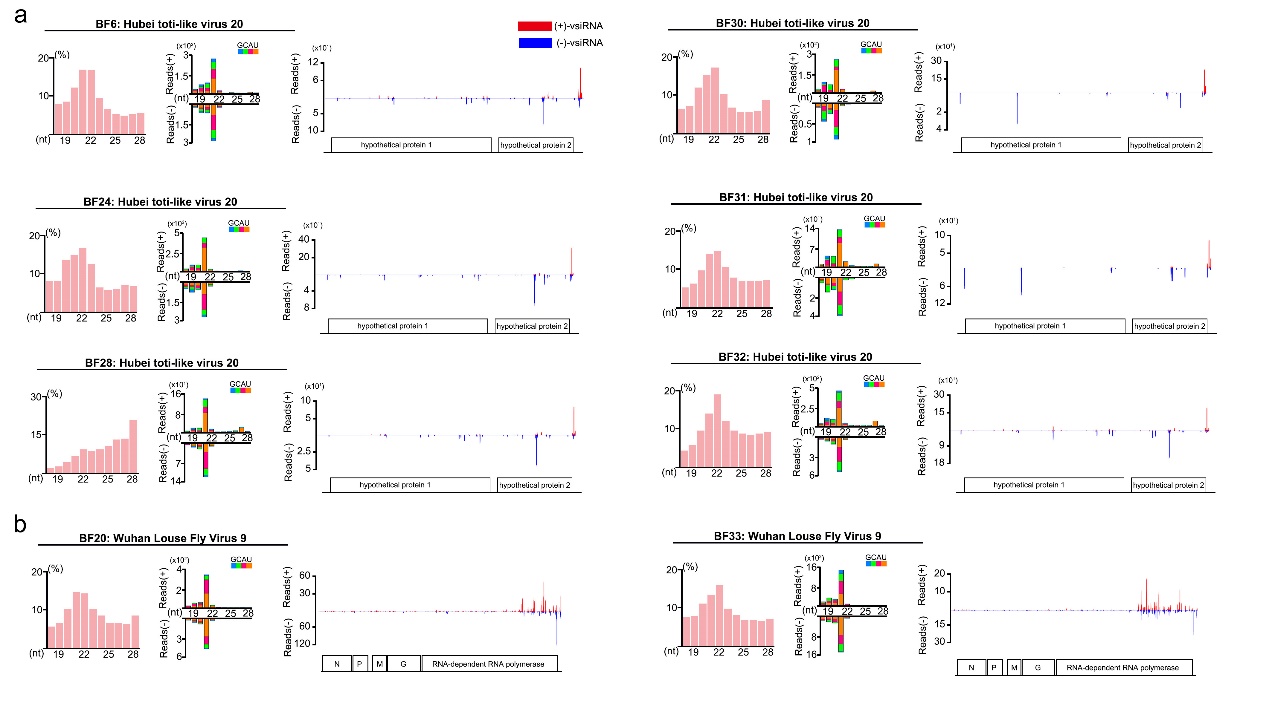
**

**Figure S4. Characterization of vsiRNAs**

**a,** The analysis of vsiRNAs derived from Hubei toti-like virus 20 in other samples.

**b,** The analysis of vsiRNAs derived from Wuhan Louse Fly Virus 9 in other samples.

Complementing the information presented in Figure 4d. The analysis methodology aligns with that described in Figure 4c. Viral genome sequences for these viruses (Hubei toti-like virus 20: KX882952; Wuhan Louse Fly Virus 9: KM817656) were obtained from the NCBI sequence database.

**Tables. S1–S3 are listed as a** **separate excel file due to their large size**

**Table S1**

**Table S1A.** Statistics of the assembled bat fly genome

**Table S1B.** Summary of sequenced reads

**Table S1C.** Mitogenome version for the species used in mitogenome assembly and taxonomic identification

**Table S1D.** Coverage depth for sequenced reads

**Table S1E.** Statistics of single base accuracy for assembled genome

**Table S1F.** Assessment of the bat fly genome completeness using CEGMA and BUSCO

**Table S1G.** Repeat content of the bat fly genome

**Table S1H.** Statistics of protein-coding genes annotation based on multiple methods

**Table S1I.** Statistics of functional annotation about protein-coding genes based on multiple databases

**Table S1J.** Basic statistics of the protein-coding genes for bat fly and related species

**Table S1K.** Genome version for the Diptera species in homology-based annotation, genome size comparative analysis, and comparative genomic analysis

**Table S1L.** Assessment of the bat fly genome annotation completeness using BUSCO

**Table S1M.** Fossil-calibrated time points used when constructing phylogenetic tree and calculating divergence

**Table S2**

**Table S2A.** GO and KEGG enrichment of expanded gene families in the bat fly

**Table S2B.** GO and KEGG enrichment of contracted gene families in the bat fly

**Table S2C.** The changed genes that associated with eye, wing, and leg development, circadian rhythms, feeding behavior, and immune

**Table S2D.** Positive selection genes in the bat fly using branch-site model analysis in PAML

**Table S2E.** Comparison of chemoreceptor genes between Bat fly, Glossina, Drosophila melanogaster and Anopheles gambiae

**Table S3**

**Table S3A.** Primers for PCR, qPCR and gene cloning related to STAR Methods

**Table S3B.** Bat fly sample information and infection virus experiments

**Table S3C.** Contents and properties of the small RNA libraries sequenced

**Table S3D.** Summary of results obtained with VirusDetect

**Table S3E.** Viruses identified in the bat fly

**Table S3F.** Viruses identified in siRNA reads

**Table S3G.** Contigs that do not meet virus identification threshold

**Table S3H.** Contigs generated by metagenomic sequencing are mapped to the known invertebrate viral genomes database

**Video S1. The process of bat fly biting mice.** Corresponding videos can be found in separate file (video format: .mp4)
